# The Many Faces of Aire in Central Tolerance

**DOI:** 10.3389/fimmu.2013.00326

**Published:** 2013-10-11

**Authors:** Martti Laan, Pärt Peterson

**Affiliations:** ^1^Molecular Pathology, Faculty of Medicine, Institute of Biomedicine and Translational Medicine, Tartu University, Tartu, Estonia

**Keywords:** Aire, thymus, Hassall’s corpuscle, thymic epithelial cells, central tolerance, chemokines, negative selection, epithelial differentiation

## Abstract

Although the role that Autoimmune Regulator (Aire) plays in the induction of central tolerance is well known, the precise cellular and molecular mechanisms are still unclear and debated. In the prevailing view, Aire serves mainly as a direct inducer of tissue-specific antigens. However, there is a growing amount of evidence suggesting that Aire modulates the differentiation program of medullary thymic epithelial cells, which may directly contribute to the negative selection of self-reactive thymocytes. In addition, Aire has been shown to regulate the expression of many intrathymic chemokines that are required for the proper localization of thymocytes and dendritic cells, and thus are potentially important for direct and indirect self-antigen presentation in the thymic medulla. Further, recent evidence suggests that the induction of certain antigen-specific regulatory T-cells that translocate to tumors and peripheral tissues can be Aire dependent and may contribute to tissue-specific tolerance. This review summarizes the current understanding of the effects of Aire on these alternative mechanisms for the induction of Aire-induced central tolerance.

The thymus is the primary lymphoid organ involved in thymocyte development and thus plays a central role in establishing immune tolerance (1). During the course of central tolerance induction, single-positive thymocytes are guided from the thymic cortex to the medulla, tested there for reactivity to self-antigens and, if they are self-reactive, either deleted or directed to become regulatory T-cells (Tregs). Impaired clonal deletion or Treg induction can lead to a breakdown of central tolerance and the development of autoimmune diseases.

## Aire Deficiency Results in Autoimmunity

An essential molecule in the induction of central tolerance is Autoimmune Regulator (Aire). The *AIRE* gene was identified by positional cloning of a locus linked to a rare disease, Autoimmune-Polyendocrinopathy-Candidiasis-Ectodermal Dystrophy (APECED) (2, 3). This syndrome usually starts during childhood with chronic mucocutaneous candidiasis, which may correlate with autoantibodies to interleukin (IL)-17 and IL-22 (4). The later stages of this disease are characterized by the presence of autoantibodies to multiple self-antigens and lymphocytic infiltration of various endocrine glands, which finally leads to autoimmune endocrine disorders (5, 6). Although the phenotype of Aire-deficient mice is considerably milder than in APECED and is dependent on the genetic background, it is also characterized by autoantibodies and autoimmune infiltrations, and thus resembles the pathological characteristics of APECED patients (7).

## Aire Deficiency Results in Defective Negative Selection

There is strong experimental evidence that Aire deficiency directly results in the defective negative selection of thymocytes. This evidence comes from transgenic mice in which most of the T-cells express T-cell receptors (TCRs) specific for a certain neo-self-antigen, such as hen egg lysosome (HEL). When this transgenic line is crossed with another transgenic line expressing HEL under the rat insulin promoter [i.e., an RIP-HEL mouse expressing HEL in thymic medullary thymic epithelial cells (mTECs) and in the pancreas], the effectiveness of eliminating autoreactive T-cells by negative selection is strictly dependent on the presence of Aire (8). This role in regulating the thymic clonal deletion of autoreactive thymocytes has also been shown for other neo-self-antigens and for different promoters and clearly indicates a role for Aire in negative selection (9, 10).

## Aire is Predominantly Expressed in MHC Class II-High, CD80-High mTECs

Several studies have expanded our knowledge of Aire and illustrate its key role in central tolerance induction. The majority of Aire signal have been shown to come from mTECs, a very specific set of cells in the thymus (11). mTECs are unique because they can express thousands of tissue-specific self-antigens that are presented to developing thymocytes and are thus associated with negative selection (12). The phenomenon, known as promiscuous gene expression, and the role that Aire plays in this process, have been covered in detail by Ucar et al. in this Research Topic of the Frontiers in Immunology. Within mTECs, Aire expression is specifically located in a subpopulation of cells characterized by the surface expression of MHC class II, and the co-stimulatory molecules CD80 and CD86 (13), indicating that, in addition to antigen cross-presentation by dendritic cells (DCs), Aire+ mTECs also have the potential for direct antigen presentation. Within these MHC class II mTECs, Aire localizes in the nuclei, forming discrete dot-like structures that resemble promyelocytic leukemia (PML) nuclear bodies (11, 14). PML bodies have been associated with several activities, including the modulation of chromatin structure, transcriptional control, DNA repair, and antiviral response (15).

In addition to mTECs, some recent studies have also identified the Aire protein in peripheral lymph nodes both in humans as well as mice (16, 17). It has been shown that the peripheral expression of Aire may contribute to the autoimmune phenotype either via its effects on T-cells as well as directly on B-cells (16, 18). Thus, in addition to its major role in the induction of central tolerance, as is covered in this review, Aire is likely to play a role in peripheral tolerance as well, as has been covered in recent reviews (19).

**Figure 1:**
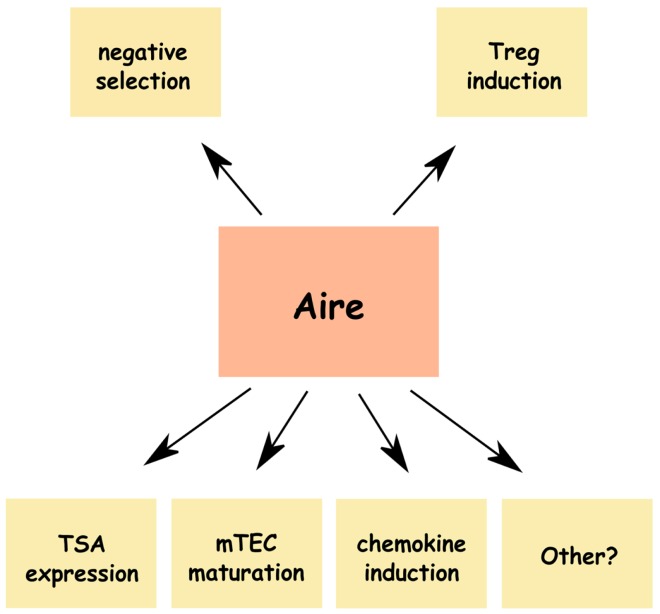
**Alternative mechanisms of Aire-induced central tolerance**. In addition to the well-described roles in tissue-specific antigen (TSA) expression and induction of negative selection Aire has been shown to affect maturation of medullary thymic epithelial cells (mTECs), expression thymic chemokines, and induction of naturally occurring Tregs, which may all contribute to the induction of central tolerance. Other possible mechanisms, such as induction of mTEC apoptosis or disturbances in thymocyte maturation have also been suggested.

Thus, in summary, it is well established that (1) the insufficient expression of Aire results in autoimmunity in both humans and mice, (2) Aire is required for negative selection, and (3) the MHC class II-high, CD80-high mTEC population is the primary source of Aire. However, the precise mechanisms that link the expression of Aire in the thymus to the effective induction of tolerance, are still widely debated and are summarized in the remainder of this review (Figure 1).

## Aire and TSA Expression

The role of Aire as a master regulator of tissue-specific antigens (TSA) was first proposed by Anderson et al. (20) and is by far the best studied and established mechanism behind Aire-induced negative selection. It is clear that Aire controls the expression of many TSAs in mTECs and that Aire-dependent expression of these TSAs leads to the negative selection of self-reactive thymocytes. These aspects of Aire have been covered in depth in recent reviews on Aire (21, 22), and also in a review on promiscuous expression by Ucar et al. in this Research Topic of the Frontiers in Immunology. However, there is also accumulating evidence that the impaired expression of TSAs is not the only mechanism behind Aire-induced central tolerance. For example, Aire-deficient mice develop a Sjögren’s syndrome-like autoimmune reaction to α-fodrin, which is a self-antigen not regulated by Aire (23). Similarly, Aire-deficient non-obese diabetic (NOD) mice develop autoimmune pancreatitis to isomerase A2, another Aire-independent self-Ag (24). Therefore, it is clear that Aire has an additional effect on T-cell selection independent of its effect on TSA expression. The precise mechanisms responsible for these additional effects of Aire, however, are still unclear and deserve further studies.

## Aire and mTEC Maturation

**Figure 2:**
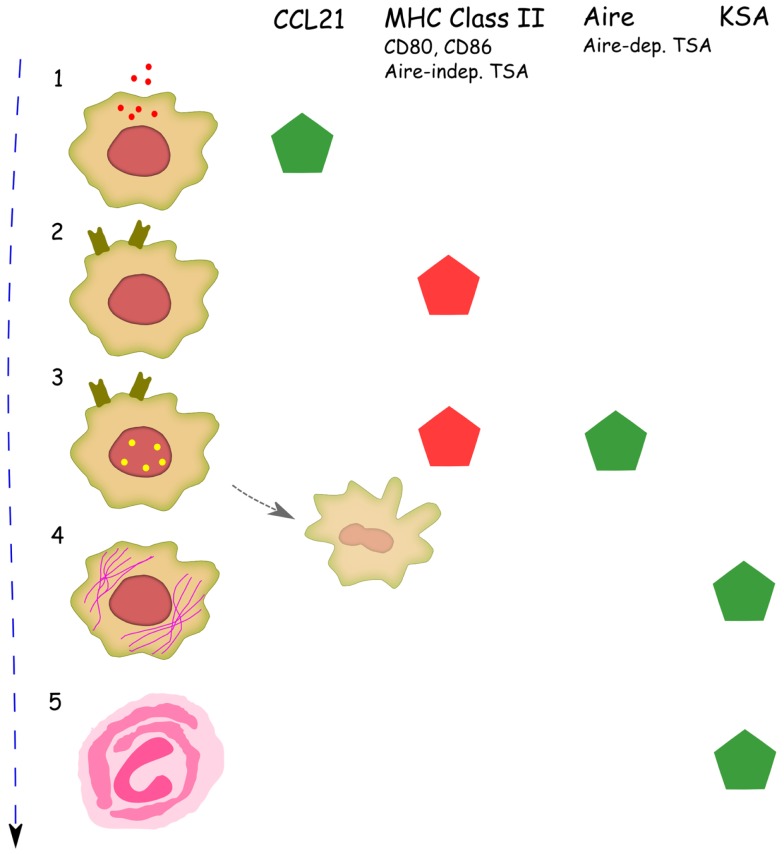
**Proposed program of mTEC differentiation and the effect of Aire on mTEC development at different stages of differentiation**. Consecutive developmental stages are indicated based on key molecules predominantly expressed at this stage: (1) MHC class II−, CCL21+*; (2) MHC class II+, Aire−; (3) MHC class II+, Aire+; (4) MHC class II-low, Aire−, keratin high; (5) Hassall’s corpuscles. Possible pathway from MHC II+, Aire+ cells to apoptosis is shown in pale. The pentagonal signs indicate presence of the specific marker at this stage of differentiation, whereas the color of a sign indicates the change of this marker in the Aire-deficient mice: green corresponds to a decrease and red to an increase in Aire KO mice. TSA stands for tissue-specific antigens and excludes keratinocyte-specific TSAs; KSA stands for keratinocyte-specific antigens. *In parallel to the developmental sequence presented here, the MHC class II−, CCL21+ mTEC stage has been suggested to follow the MHC class II+, Aire+ stage of development (50).

During maturation, mTECs must pass through several developmental stages that are characterized by the expression of a few key proteins that are related to specific functions at that particular stage of development (Figure 2). The classical subpopulations are composed in consecutive order of the (1) MHC class II-low, CD80-low, Aire-mTECs, which are considered to be an immature, highly proliferating population, that is already committed to the mTEC lineage; (2) MHC class II-high, CD80-high, Aire-mTECs, a subpopulation that is already capable of expressing many (Aire-independent) TSAs and directly presenting them to developing thymocytes; and (3) MHC class II-high, CD80-high, Aire+ mTECs. This last cell population consists of matured, post-mitotic mTECs that express a wide variety of TSAs, including the ones under control of Aire (25, 26). Until quite recently, it was widely accepted that these post-mitotic Aire+ mTECs represented the final stage of mTEC maturation. More recent data, however, suggest that the maturation of mTECs may extend beyond the Aire+ stage, and Aire in fact may modulate several different aspects of mTEC differentiation. The first indications that Aire may alter mTEC differentiation came from studies by Gillard et al. and Dooley et al., that characterized the staining patterns of UEA1, Keratin (K)-5/K8/K14, and p63 in Aire-deficient thymi. These authors observed changed proportions of stellate versus globular mTECs and more frequent thymic cysts in Aire-deficient thymi. Although these findings were only apparent after careful analysis, and their functional significance is difficult to evaluate, they clearly suggest that the loss of Aire alters the basic parameters that determine mTEC morphology and maturation stage (27, 28). In addition, a study by Milicevic et al. looked at the ultrastructure of mTECs of Aire-deficient mice using electron-microscopy and found profound changes in all subpopulations of mTECs (29). Rather strikingly, all mTECs in Aire-deficient mice, regardless of their differentiation stage, showed profound ultrastructural changes. These changes were collectively characterized as signs of activation and increased intracellular traffic. Thus, although mTECs classified by either electron-microscopy or functional surface markers are not directly comparable, this ultrastructural study suggested that the effect of Aire on mTEC maturation is not restricted to the final (i.e., Aire+) cells, but rather covers all mTEC subpopulations.

Direct evidence suggesting that mTECs survive and further differentiate after the Aire+ stage originated from recent studies by Yano et al., Nishikawa et al., and Wang et al., all of which used different reporter mice to follow the faith of Aire+ cells (30–32). These studies demonstrated that Aire+ mTECs develop further into MHC class II-low, CD80-low, Aire-mTECs, and thus lose their unique property of direct TSA presentation in parallel with the loss of Aire (31, 32). In addition, the loss of another unique property of Aire+ cells, promiscuous TSA expression, was indicated by the down-regulation of many Aire dependent as well as Aire-independent TSAs in these post-Aire mTECs (32). Instead, post-Aire cells upregulated the expression of keratins and thus came to resemble keratinocytes, at least in terms of the gene-expression pattern (30, 32). Finally, it was shown that the post-Aire cells lose their nuclei and merge to become Hassall’s Corpuscles (HC), well known but poorly characterized concentric structures in thymic medullary areas (30, 32). Although the precise function of these structures is unknown, they may have the potential to induce Treg development through expression of TSLP (33). In addition, it has been shown that intrathymic expression of some keratinocyte-specific TSAs, including the expression of the well-known pemphigus-related autoantigens, desmoglein-1 and -3, is clearly restricted to HCs (32, 34–36). Thus, it is plausible that these post-Aire structures contribute to the induction of central tolerance by negative selection of keratinocyte-reactive thymocytes as well as by induction of keratinocyte-specific Tregs.

The lack of Aire seems to block the maturation of mTECs, resulting in the accumulation of MHC class II+, CD80+, truncated Aire+ mTECs, and in a severe reduction in the expression of many keratins and the numbers of HCs (28, 31, 32) (Figure 2). The reduction in the expression of keratinocyte-specific proteins in Aire-deficient thymi involves also desmoglein-3 and results in the altered selection of desmoglein-3-specific T-cells and defective tolerance against desmoglein-3 (37). Thus, it has been formally demonstrated that through its effect on mTEC differentiation, Aire can promote tolerance at least against this specific keratinocyte-related TSA. Whether, and to what extent, the broad effect of Aire on gene expression is regulated through mTEC differentiation rather than direct transcriptional activity, remains to be determined in future studies.

## Aire and Chemokine Expression

**Figure 3:**
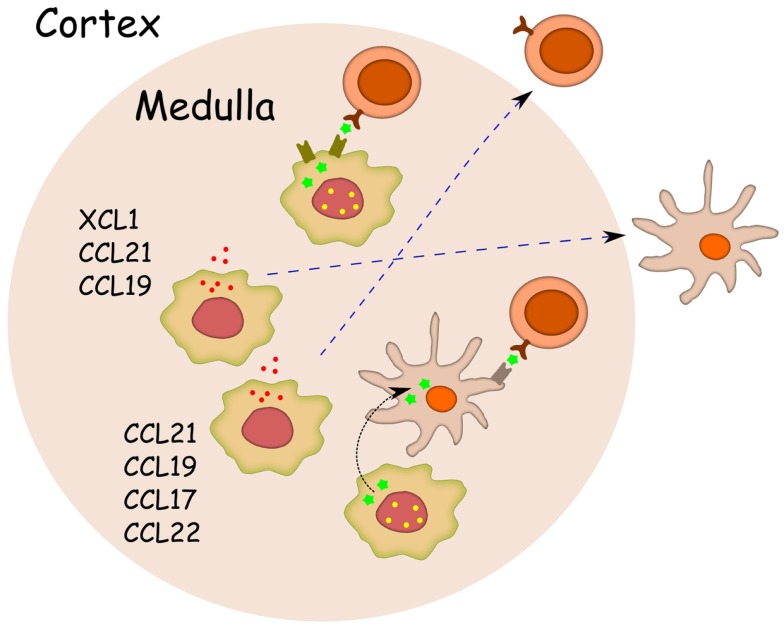
**Aire regulates chemokines required for proper localization of thymocytes and dendritic cells**. For effective direct antigen presentation (shown as green pentagons) from mTEC to thymocyte, the positively selected single-positive thymocytes need to migrate from cortex to medulla. For effective indirect antigen presentation both, the positively selected thymocytes and the DCs, need to migrate to medulla. Only the key chemokines (shown as red particles) known to mediate the migration of single-positive thymocytes or DCs, and also known to be regulated by Aire (shown as yellow dots) are listed.

The negative selection of developing thymocytes is also dependent on coordinated migration through distinct thymic niches, that provides timely interactions with mTECs and DCs (38). Cell–cell contact with TSA-expressing mTECs requires that the positively selected thymocytes migrate from the thymic cortex to the medulla, whereas indirect TSA presentation is likely to be dependent on the physical proximity of thymocytes, mTECs and DCs (39) (Figure 3). The ligands of two chemokine receptors, CCR7 and CCR4, have been previously associated with thymocyte migration to the site of negative selection. Both receptors are predominantly expressed on double positive (DP) and single-positive (SP) CD4-thymocytes (40–42), while the ligands for CCR7 and CCR4 are produced predominantly in the thymic medulla (40, 41, 43–45). The importance of CCR7 ligands in thymocyte development has been further highlighted in the CCR7-deficient mouse, in which the impaired intrathymic migration of thymocytes resulted in a delay of mature T-cell emigration and a leakage of premature T-cells (45). Significantly, a lack of CCR7 caused defective induction of central tolerance and the presence of multiple autoantibodies and autoimmune infiltrations in many peripheral organs, resulting in a phenotype almost identical to that of Aire-deficient mice (46, 47). In turn, the importance of Aire in the regulation of thymic chemokine expression has been reported in many studies. First, microarray analysis of sorted mTECs has demonstrated the down-regulation of several chemokines in the Aire-deficient mouse (10). Second, the effect of Aire on CCR4 and CCR7 ligand expression has been further validated with both the up- and down-regulation of Aire (48, 49). Third, the delay of thymocyte emigration (in a manner similar to that in CCR7-deficient mice) is also present in the Aire-deficient mice (48). Notably, the highest expression of CCR7 ligands, although Aire dependent, occurs in the MHC class II-low mTECs, i.e., in a population not expressing Aire (48, 50). Further, during mouse ontogeny, CCL21+ (i.e., CCR7 ligand-producing) cells appear after the appearance of Aire+ cells (50). It has therefore been proposed that MHC class II-, CD80−, CCL21+ mTECs represent the post-Aire population (50). However, we have measured the levels of multiple chemokines directly in the post-Aire population in the Aire-reporter mouse, and found a clear down-regulation of all measured chemokines in these post-Aire cells (32). Therefore, although the precise developmental sequence is still clearly under debate, we feel that the MHC class II-, CD80− population prior to the induction of Aire is a more likely source of CCR7 ligands (Figure 2). It remains unclear, however, how the expression of Aire in one cell can influence another mTECs in a less mature stage. In addition to possible direct paracrine signaling from mTEC to mTEC, another possibility involves cross-talk between mTECs and thymocytes, which do not receive all appropriate signals from the mTECs under Aire-deficient situation, resulting in improper signaling from thymocytes back to immature mTECs. This effect of Aire on mTEC maturation and differentiation before and after Aire expression is in agreement with the ultrastructural changes observed during all stages of mTEC development that have been discussed above (29).

In addition to its effect on thymocyte migration, Aire expression in mTECs has been shown to regulate the chemokines responsible for DC migration. Although not characterized in the thymus, peripheral DCs bear the CCR7 receptor, which is required for their proper localization within lymph nodes (51). In addition, it has been shown by Lei et al. that thymic DCs express the receptor for the chemokine XCL1, which is specifically expressed by mTECs in an Aire-dependent fashion, and is required for the proper localization of DCs to the cortico-medullary junction (49). Thus, there is evidence that Aire-dependent chemokines are required for thymocyte and DC migration to the location where antigen (cross)-presentation and negative selection are likely to occur (Figure 3). In fact, altered cross-presentation in *Aire* KO mice has previously been demonstrated (52), although the roles of specific chemokines in this process are still unclear.

## Aire and Treg Induction

There is an increasing amount of direct evidence that, in addition to their proposed role in negative selection, mTECs contribute to central tolerance by inducing naturally occurring Tregs (53, 54). Accordingly, since the characterization of an autoimmune phenotype in Aire-deficient mice, a significant number of studies have focused on the potential role of Aire in thymic Treg induction. However, there was no major change in either Treg numbers or function in transgenic models (RIP-HEL, RIP-mOVA), in which the negative selection of neo-self-antigen-specific thymocytes was clearly Aire dependent (8–10). Likewise, the lack of Aire had no major effect on TCR usage by Foxp3+ Tregs, in a study where the effect of Aire on Treg TCR repertoire was assessed in a mouse model with restricted TCR repertoire (55). Thus, initial studies that looked at the total numbers and/or function of Tregs, indicated that defects in central tolerance in Aire-deficient mice are not Treg related. Nevertheless, a recent study showed that Aire expression is required for the intrathymic production of tumor-specific Tregs in a mouse model of oncogene-driven prostate cancer (56), and clearly demonstrated a role for Aire in induction of a specific population of naturally occurring Tregs. Thus, this study demonstrates directly that the key role of Aire in central tolerance is not limited to its effect on negative selection but also includes its effect on thymic Treg induction. Future studies will determine, whether this intriguing effect of Aire extends to a significant pool of TSA-specific Treg induction and, if so, what mechanisms cause this effect.

## Other Plausible Mechanisms Behind Aire-Induced Central Tolerance

There are also a number of studies indicating that in addition to the mechanisms covered above, Aire may influence other basic mechanisms, directly in mTECs or indirectly in other thymic cells which, at least hypothetically, may contribute to defects in negative selection.

Thus, Aire may play a role in the induction of apoptosis, as the over-expression of Aire results in the induction of apoptosis in several *in vitro* cell-lines (25, 57, 58). Based on these data, it has been proposed that the absence of this effect is responsible for the increased numbers of MHC class II+ mTECs observed in Aire-deficient mice and that Aire-induced apoptosis may facilitate the cross-presentation of TSAs expressed by these apoptotic cells to the thymic DCs (25, 28). As the apoptotic processes in the thymus are very dynamic and thus difficult to monitor, this attractive hypothesis has not yet been validated *in vivo*. It is, however, completely plausible that, in addition to the maturation of post-Aire mTECs and HCs, a subpopulation of Aire+ mTECs are directly guided to undergo apoptosis and are then quickly removed by resident macrophages (Figure 2).

In addition, a report by Li et al. shows that the development of SP CD4-thymocytes is blocked at the transition from SP3 to SP4 in Aire-deficient mice (59). Although this may be due to imperfect cross-talk between defectively matured Aire-deficient mTECs and developing thymocytes, or to insufficient cell–cell contact as a result of reduced chemokine expression, neither of these possibilities has been formally tested. Additionally, the functional consequences of this phenomenon remain unknown.

In summary, although the effects of Aire on central tolerance are well established, the cellular and molecular mechanisms are still unclear. Along with the better-understood effects on TSA expression, Aire can also alter the differentiation program of mTECs, regulate the expression of thymic chemokines, contribute to specific Treg induction, and induce mTEC apoptosis. It remains to be determined, however, what extent these alternative mechanisms contribute to the autoimmune phenotype observed in Aire-deficient mice and APECED patients.

## Conflict of Interest Statement

The authors declare that the research was conducted in the absence of any commercial or financial relationships that could be construed as a potential conflict of interest.
